# Roles of AMP-Activated Protein Kinase (AMPK) in Mammalian Reproduction

**DOI:** 10.3389/fcell.2020.593005

**Published:** 2020-11-19

**Authors:** Weina Yang, Lingjuan Wang, Fengli Wang, Shuiqiao Yuan

**Affiliations:** Institute of Reproductive Health, Tongji Medical College, Huazhong University of Science and Technology, Wuhan, China

**Keywords:** AMPK, GnRH, reproduction, spermatogenesis, transcription

## Abstract

Reproduction is an energy demanding function and only take place in case of sufficient available energy status in mammals. Metabolic diseases such as anorexia nervosa are clinically associated with reduced fertility. AMP-activated protein kinase (AMPK), as a major regulator of cellular energy homeostasis, is activated in limited energy reserves to ensure the orderly progress of various physiological activities. In recent years, mounting evidence shows that AMPK is involved in the regulation of reproductive function through multiple mechanisms. AMPK is likely to be a metabolic sensor integrating central and peripheral signals. In this review, we aim to explore the preclinical studies published in the last decade that investigate the role of AMP-activated protein kinase in the reproductive field, and its role as a target for drug therapy of reproductive system-related diseases. We also emphasized the emerging roles of AMPK in transcriptional regulation of reproduction processes and metabolisms, which are tightly related to the energy state and fertility of an organism.

## Introduction

AMP-activated protein kinase (AMPK) is a heterotrimeric complex composed of a catalytic α-subunit and two regulatory subunits: β and γ. The most well-known physiological function of AMPK is to act as an energy sensor to maintain metabolic homeostasis (Hardie, 2007). In response to decreased ATP/AMP ratio or glucose starvation, the liver kinase B1 (LKB1) phosphorylates the threonine-172 residue of AMPK and mediates its activation through canonical and non-canonical mechanisms. These two activation mechanisms occur in the cytoplasm and lysosome, respectively (Gonzalez et al., 2020). Furthermore, hormones and DNA damage can also trigger AMPK activation by the Ca^2+^/calmodulin-dependent kinase, CaMKK2 (Gonzalez et al., 2020). After activated, AMPK switches off ATP-consuming pathways such as protein synthesis, glycogenolysis, and lipogenesis, and turns on ATP-generating pathways such as fatty acid oxidation, glycolysis, and autophagy by regulating downstream factors, thereby ensuring nutrient supply (Hardie, 2007).

It is known that mammalian reproduction is an energy-consuming process that occurs when there is adequate nutrition (Dupont et al., 2014). Metabolic disorders are clinically associated with fertility decline. For instance, polycystic ovary syndrome (PCOS) is a common reproductive disorder that causes infertility in women of childbearing age often accompanied by insulin resistance and hyperandrogenemia (Diamanti-Kandarakis, 2008). Besides, anorexia nervosa or obesity may cause impaired ovarian function and spermatogenesis (Group, 2006; Pasquali, 2006; Mah and Wittert, 2010). Therefore, the relationship between reproduction and energy metabolism has attracted extensive attention. The properties of AMPK as an energy sensor allow it to couple the energy status of the body to reproductive function (Duval, 2011). Hypothalamic AMPK is central to energy homeostasis (Huynh et al., 2016). Both anorectic signals (leptin, insulin) and orexigenic signals (ghrelin, neuropeptide Y) affect energy intake and energy expenditure through the regulation of hypothalamic AMPK activity (Minokoshi et al., 2004), thereby ensuring the orderly progress of various physiological activities. During nutrient starvation, AMPK, as a catabolic enzyme, promotes cellular catabolism and augments energy production through the inhibition of mTOR (mammalian target of rapamycin) by directly phosphorylating the TSC2 (tumor suppressor Tuberous Sclerosis Complex 2) and RAPTOR. Under nutrient-rich conditions, the inhibition of mTOR is diminished and the whole-body energy balance is positive, it is conducive to reproduction (Gonzalez et al., 2020). In addition to the above indirect effects on reproduction, AMPK in the hypothalamus and pituitary is able to directly regulate gonadal steroid hormones production and affect fertility in response to peripheral metabolic signals (Tulipano, 2020). However, the global physiological actions of AMPK in reproduction include not only the acute response of AMPK activation through directly phosphorylating downstream metabolic enzymes or regulating the hypothalamic pituitary gonadal (HPG) axis, but also AMPK-mediated transcriptional events via affecting the interaction between transcription regulators (TFs) and their DNA recognition sites.

In this review, we aim to explore the preclinical studies published in the last decade that investigate functions of AMP-activated protein kinase in the reproductive field, and its role as a target for drug therapy of reproductive system related diseases. We also discussed the emerging roles of AMPK in transcriptional regulation of reproduction processes and metabolisms, which are tightly related to the energy state and fertility of an organism.

## AMPK Is Involved in the Modulation of the Hypothalamic Pituitary Gonadal (HPG) Axis

The HPG axis is of vital importance in mammalian reproductive system (Schneider, 2004). The secretion and gene transcription of the pituitary gonadotropins luteinizing hormone (LH) and follicle-stimulating hormone (FSH) are driven by pulsatile release of gonadotropin-releasing hormone (GnRH) from neurons in the hypothalamus. Gonadotropins control gonadal steroid hormones production and in turn sex hormones exert feedback regulation on GnRH, LH and FSH synthesis and secretion (Burger et al., 2004). In response to energy insufficiency, the bodies usually choose to reduce all the dispensable energy expenditure in mammals. Therefore, GnRH pulsatility was suppressed, and fertility decreased to allow energy accumulation for individual survival. That is to say, there must be some signaling pathways in the center that serve as a bridge to link the diminished energy reserves with the reproductive neuroendocrine axis. In fact, hypothalamic AMPK activity is tightly related to feeding and thermogenesis, thus controlling the energy homeostasis of the organism (Lim et al., 2010; Wang and Cheng, 2018). It is worth noting that AMPK is highly expressed in those nuclei associated with reproductive control in the hypothalamus, such as the paraventricular and arcuate nuclei (Torsoni et al., 2016). Hence, AMPK pathways in the hypothalamus are likely to mediate the effects of peripheral signals on reproductive function. Kisspeptin, a protein encoded by the *Kiss1* gene, can promote GnRH secretion and affect puberty after binding with its receptor G-protein-coupled receptor-54 (GPR54) (Seminara et al., 2003; Pinilla et al., 2012; Herbison, 2016). Evidence is accumulating that AMPK activation inhibits GnRH release in GT1-7 cell, a mouse immortalized hypothalamic GnRH neuron (Coyral-Castel et al., 2008; Wen et al., 2008, 2012). On the one hand, AMPK activation induced by adiponectin represses the promoter activity of the *Kiss1* gene via inhibition of the translocation of specificity protein-1 (SP1) from the cytoplasm to the nucleus and subsequently influences GnRH secretion (Wen et al., 2012). On the other hand, the trafficking and exocytosis of secretory vesicles is ATP-dependent (Nagiec et al., 1995). As a consequence, it is plausible that AMPK may be activated to reduce GnRH secretion to increase ATP levels in limited fuel availability. However, these experiments were carried out in immortalized cells while GnRH neurons themselves do not express *Kiss1* (Pinilla et al., 2012), so the reliability of these results needs to be further confirmed. In previous years, two clinical studies have observed that metformin treatment at early stage could delay menarche and ameliorate metabolic disorder in girls with precocious pubarche (Ibanez et al., 2011a,b). A paper published in 2018 documented that the regulatory effect of AMPK activation on adolescent metabolic control under negative energy balance was achieved by reducing kisspeptin expression in hypothalamic KISS1 neurons (Roa et al., 2018). Conditional deletion of the AMPKα1 subunit in Kiss1 cells largely prevented the delay in puberty onset caused by malnutrition (Roa et al., 2018). Other AMPK subunits may be involved in the partial compensation of the lack of AMPKα1 as the AMPKα2 catalytic subunit in Kiss1 neurons has been reported to be responsible for the reproductive adaptations to acute metabolic distress (Torsoni et al., 2016).

**FIGURE 1:**
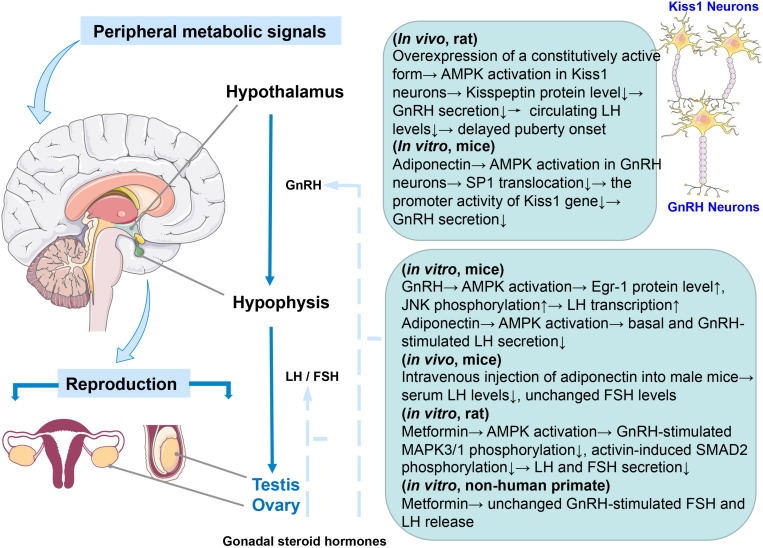
Graphical summary of the effect of AMPK activation on the HPG axis. Species and *in vivo* or *in vitro* experiments are notified.

Notably, the different subunits of AMPK are also expressed in the pituitary (Tosca et al., 2011) and the role of pituitary gonadotroph cells as a metabolic sensor to integrate energy status with fertility has been known (Duval, 2011). Clinically, PCOS is characterized by elevated LH to FSH ratios, but metformin treatment can reduce serum LH concentration in PCOS women (Velazquez et al., 1994; Genazzani et al., 2004; Oride et al., 2010). It is likely that AMPK activation mediates the effect of metformin on pituitary gonadotropin-secreting cells (Duval, 2011). In mouse LβT2 cells (mouse pituitary cell), inhibition of AMPK activity or reduction of AMPK levels prevented GnRH-stimulated LH gene transcription (Andrade et al., 2013). Interestingly, Andrade et al. reported that AMPK effects are mediated at least in part by diminishing the EGR-1 protein level (EGR-1 is a transcription factor required for LH expression and induced to synthesize in response to GnRH) (Andrade et al., 2013). However, this outcome is contrary to that of Lu et al. (2008) who found that AMPK activation by adiponectin reduces GnRH-stimulated LH secretion, and this repression can be mimicked by AICAR (5-aminoimidazole-4-carboxamide riboside), an activator of AMPK. In rat pituitary cells, metformin-induced AMPK activation strongly decreases LH and FSH in response to GnRH and FSH release induced by activin via inhibiting the MAPK3/1 and SMAD2 signaling pathways, respectively (Tosca et al., 2011). Subsequently, these alterations in gonadotropin secretion would likely lead to changes in steroid synthesis in the gonad. Unlike previous studies using rodent cells, metformin can only inhibit basal, but not GnRH-stimulated FSH secretion, without altering LH release in non-human primate pituitary cells *in vitro* (Vazquez-Borrego et al., 2018; Figure 1). These inconsistent conclusions may be due to differences in species or experimental methods, indicating that further work is required to establish the viability of exploring the effect of AMPK on the HPG axis *in vivo* and *in vitro*.

## AMPK in Male Reproduction

It is well known that the Liver Kinase B1 (LKB1), located upstream of AMPK, regulates AMPK activity (Lizcano et al., 2004). In humans, LKB1 gene mutation usually results in Peutz-Jeghers syndrome (PJS) and male infertility (Ulbright et al., 2007). Likewise, loss of LKB1 signaling in mice affects the release of mature spermatids into the seminiferous tubules (Denison et al., 2011). Additionally, genetically deleted testis-specific serine kinase (TSSK) family genes, which also belong to the AMPK branch, leading to male infertility due to haploinsufficiency (Xu et al., 2008). Thus, the fact that AMPK-related genes’ defects induce a reduction in male fertility and the widespread expression of AMPK in various testicular cells suggests its essential role in male reproduction. We will focus on the functions of AMPK in male gonadal somatic cells and spermatogenesis to summarize as following sections.

### The Effect of AMPK on Male Gonadal Somatic Cells

Male gonadal somatic cells contain two types of cells, Sertoli and Leydig cells. Sertoli cells protect and nourish developing germ cells, and the cell junction between Sertoli cell formed near the basement membrane of seminiferous epithelium constitutes the blood-testis barrier (BTB), providing a suitable microenvironment for spermatogenesis and its maturation. Additionally, Leydig cells can synthesize and secrete androgen to promote spermatogenesis (Gerber et al., 2016). Taken together, spermatogenesis is inseparable from the normal function of Sertoli and Leydig cells. Tanwar et al. (2012) reported that conditional deletion of LKB1 in Sertoli cells of mice resulted in germ cell loss and Sertoli cell-only seminiferous tubular phenotype, which is similar to that of human PJS patients (Ulbright et al., 2007). Some seminal researches have previously shown that LKB1 (Baas et al., 2004) and its downstream effector AMPK are responsible for establishing apicobasal cell polarity (Forcet et al., 2005; Lee et al., 2007) and AMPK activation reinforces tight junction function through directly phosphorylating its substrates in apical junctional complexes (Lee et al., 2007; Zhang et al., 2011; Shiomi et al., 2015; Aznar et al., 2016) or driving key epithelial transcription factors (Sun et al., 2017). Thus, it is no surprise that the LKB1 knockout mice also show defects in Sertoli cell polarity and testicular junctional complexes, which indicates the involvement of AMPK-mediated mTOR inhibition in this biological process (Tanwar et al., 2012). It is well known that the maintenance of Sertoli cell polarity and their tight junction, an integral part of the BTB, are essential for male germ cell development and spermatogenesis. The barrier functions as a “fence” to prevent the diffusion of harmful substances and the escape of sperm antigen. Autoimmune orchitis is one of the diseases caused by barrier dysfunction (Perez et al., 2012). However, the AMPK-mediated epithelial barrier repair in a pulmonary microvascular endothelial cell wound healing model makes it a possible drug target (Creighton et al., 2011). To date, the protective effect of metformin-induced AMPK activation on epithelial barrier dysfunction in different tissues has been discovered in succession (Seo-Mayer et al., 2011; Spruss et al., 2012; Liu et al., 2014; Xue et al., 2016; Chen et al., 2018). Sertoli cells, which are closely linked to germ cells, engulf the extra cytoplasm of elongated spermatids before their release and play a key role in shaping the head of the spermatozoon (Sakai and Yamashina, 1989). Loss-function of AMPKα1 in mouse Sertoli cells causes a decrease in male fertility with abnormal sperm head morphology due to destroyed tight junction between Sertoli cells and germ cells (Bertoldo et al., 2016). In addition, AMPK activity can influence the intracellular metabolism and cell proliferation of Sertoli cells. Galardo et al. (2007) found that stimulation of rat Sertoli cells with AICAR treatment increase lactate production through several biochemical mechanisms, including promoting glucose transport and the conversion of pyruvate to lactate as well as improving lactate export to germ cells, suggesting a role for AMPK modulating the nutritional function of Sertoli cells. Specific deletion of AMPKα1 in Sertoli cells showed an increase in lipid droplets (Bertoldo et al., 2016), which is consistent with cytoplasmic vacuolization of Sertoli cells in human PJS patients (Ulbright et al., 2007). Paradoxically, there was no change in the number of Sertoli cells when AMPKα1 inactivated *in vivo*, but inactivation of AMPKα1 *in vitro* promoted Sertoli cell proliferation (Bertoldo et al., 2016). This is supported by a series of other *in vitro* experiments. For instance, administration of AMPK activator metformin to human and murine organotypic testes cultured *in vitro* led to a decrease in the number of Sertoli cells (Tartarin et al., 2012b). When pregnant mice or sexually immature chicken were exposed to metformin, loss of testis weight, and decreased proliferative activity of Sertoli cells can be observed (Tartarin et al., 2012b; Faure et al., 2016). These studies suggested that AMPK activation could inhibit cell division during Sertoli cell development. Another intriguing observation was that despite the number of aforementioned Sertoli cells of chicken was reduced, the population of spermatogonia was unchanged (Faure et al., 2016). Besides, the AMPK α1 subunit is expressed in somatic cells (Sertoli and Leydig cells) (Tartarin et al., 2012a). AMPK α1 KO male mice show a decrease in fertility, with an increased mean number of Leydig cells (Tartarin et al., 2012a). The smooth endoplasmic reticulum activity was enhanced, and testosterone synthesis increased in AMPK α1 KO testes. Owing to the negative feedback, the serum LH and FSH concentrations became lower (Tartarin et al., 2012a). In rat Leydig cells, the AMPK activator resveratrol reduces the secretion of testosterone by affecting the transport of cholesterol into mitochondria and conversion of progesterone into androstenedione (Svechnikov et al., 2009). This is in line with androgen reduction in organotypic testis cultured *in vitro* with metformin stimulation (Tartarin et al., 2012b). Therefore, the above-mentioned data strongly suggest that AMPK inhibits the production of androgen in Leydig cells and thus affects spermatogenesis.

### The Function of AMPK in Spermatogenesis

Spermatogonia undergo two rounds of meiosis and subsequent morphological change to develop into a spermatozoon during mammalian spermatogenesis (Mruk and Cheng, 2015). Ejaculated spermatozoa do not yet have the ability to complete egg fertilization in mammals. They need to go through some cellular processes in the female genital tract, including capacitation and acrosome reaction, and eventually fertilize the oocytes (Castillo et al., 2019). At this time, spermatozoa have already lost corresponding organelles to allow transcription and translation of genes, so the acquisition of these physiological abilities must rely on the modification of existing proteins (Castillo et al., 2019). AMPK and its phosphorylated form are expressed in a variety of mammalian sperm and mainly localized at the acrosome and at the mid-piece of flagellum (Martin-Hidalgo et al., 2018). Spermatozoa motility depends on flagellum beating, and mitochondria exist in the middle of the flagellum to provide energy for the movement (Gu et al., 2019). AMPK activity has been reported to be essential for spermatozoa motility. For example, AMPK inhibitor compound C (CC) treatment reduces a potent decrease in the number of mobile spermatozoa, and those remnant active spermatozoa move with significantly lower speed, but the use of CC itself does not impair sperm viability (Hurtado de Llera et al., 2012). Interestingly, AMPK activator A769662 does not improve spermatozoa motility (Hurtado de Llera et al., 2015), indicating that AMPK activity needs to fluctuate within a specific physiological range, and excessive inhibition or activation is not conducive to maintaining optimal sperm motility. In addition, the AMPKα1 knockout mice presented a reduced number of pups per litter due to decreased mitochondria number and their abnormal arrangement along microtubules in KO spermatozoa (Tartarin et al., 2012a). Similarly, specific deletion of Tssk2 in mice can also affect spermatozoa motility since it phosphorylates a protein of the axoneme central apparatus in the flagellum (Zhang et al., 2008). These studies raise the possibility that AMPK phosphorylates specific proteins in the flagellum, thereby influencing sperm motility.

Mitochondrial function is a prerequisite for sperm to achieve flagellum swing (Gu et al., 2019). The mitochondrial membrane potential (ΔΨm) is generally used to reflect mitochondrial function (Sakamuru et al., 2016). Inhibition of AMPK activity in boar spermatozoa by CC treatment resulted in instability of ΔΨm and plasma membrane lipid disorganization (Hurtado de Llera et al., 2013). Plasma membrane disorder is most likely to occur at the apical part of the acrosome, where a majority of phosphorylated AMPK protein accumulates, leading to impaired acrosomal integrity (Hurtado de Llera et al., 2013). Male germ cells will encounter various metabolic stresses in the process of transit through the female genital tract or semen cryopreservation (Huang et al., 2015). In this case, the integrity of sperm structure and intracellular adaptive physiological responses, such as AMPK regulating cell metabolism when ATP is limited, are required for successful fertilization. A study has reported that intracellular messengers Ca^2+^and cAMP, as well as PKA, PKC, and CaMKKα/β signaling pathways are involved in the activation of AMPK in boar spermatozoa in response to a variety of physiological or pharmacological stimuli (Hurtado de Llera et al., 2014).

**FIGURE 2:**
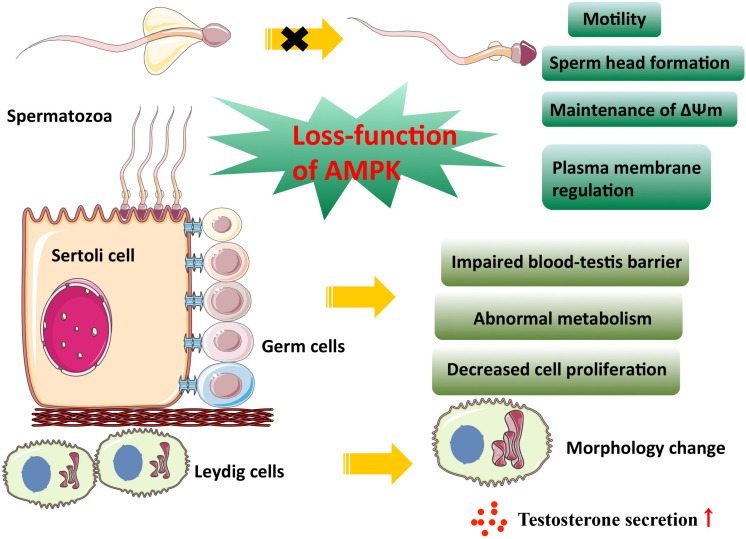
AMPK activity in male reproduction. This figure summarizes the effect of AMPK on male gonadal somatic cells and spermatogenesis. As is shown above, the loss-function of AMPK leads to the impaired physiological function of somatic cells and spermatozoa.

Data shows a decline in sperm quality among men of different races (Levine et al., 2017; Mishra et al., 2018), and in order to improve fertility, semen cryopreservation has become a fundamental part of assisted reproductive techniques (ARTs) during recent years. However, the freeze-thaw process has detrimental effects on the biological functions of spermatozoa. It can decrease the percentage of mobile spermatozoa and dramatically increase reactive oxygen species (ROS) generation and the percentage of apoptotic-like sperm cells (Shabani Nashtaei et al., 2017). Since sperm membranes are rich in polyunsaturated fatty acids (Dandekar et al., 2002), they are very susceptible to lipid peroxidation. Although there are different kinds of antioxidant enzymes in semen to protect sperm from lipid peroxidation (Aitken et al., 1996; Gadea et al., 2004), the freeze-thaw process has detrimental effects on enzyme activity and decreases the scavenging capacity of seminal plasma (Lasso et al., 1994; Bilodeau et al., 2000). AMPK activation of chicken sperm before cryopreservation enables sperm to store more ATP, which helps to restore antioxidant enzyme activity to remove ROS and limit lipid peroxidation, thus improving sperm motility and acrosome reaction, and ensuring a better quality of cryopreserved sperm (Nguyen et al., 2015). Moreover, considering that cryopreservation-induced ROS can generate a significant DNA damage in some genome regions (Thomson et al., 2009; Aitken and De Iuliis, 2010) and successful fertilization requires the sperm normal DNA integrity (Zini et al., 2008), it is important to use antioxidants to reduce oxidative DNA damage (Agarwal et al., 2004). In human frozen-thawed sperm, AMPK activator resveratrol (RSV), a known antioxidant, alleviated oxidative stress induced by cryopreservation and at least partially restored some functional properties of sperm, while CC supplementation showed an opposite effect (Shabani Nashtaei et al., 2017). Subsequent published paper showed that the addition of RSV could protect sperm DNA integrity and key paternal transcripts from the adverse effects of cryopreservation by enhancing the activity of AMPK (Shabani Nashtaei et al., 2018). The protective mechanism of AMPK activation on cryopreservation-induced DNA damage in human spermatozoa still remains obscure, which may be associated with the role of AMPK in autophagy (Li et al., 2017) or DNA damage repair (Hashimoto et al., 2018; Chen et al., 2020). These results indicates that AMPK is a drug target for optimizing sperm cryopreservation protocol and AMPK activity is crucial to protect sperm from lipid peroxidation and ROS (Figure 2).

## AMPK in Female Reproduction

Mammalian oogenesis begins in the fetus. The primordial germ cells proliferate and differentiate into oogonia, and undergo the first meiosis, reaching diplotene at birth known as a germinal vesicle (GV). When the estrus cycle arrives, the oocytes continue the first meiotic division, including germinal vesicle breakdown (GVBD), emitting the first polar body, and stopping at the middle stage of the second meiosis which won’t be completed until successful fertilization (Sathananthan et al., 2006). Both folliculogenesis and embryo development require energy. As a central regulator of energy homeostasis, AMPK is closely associated with female reproduction, including follicular development, granulosa cell proliferation, and pregnant regulation.

### Role of AMPK in Follicular Development

The mammalian ovary is a heterogeneous organ containing follicles at various developmental stages, and its principal function is the release of the mature oocyte for fertilization (McGee and Hsueh, 2000). Although many follicles exist in the ovary, most of them are primordial follicles, which consist of an immature oocyte and several surrounding flattened somatic cells called primordial follicle granulosa cells (McGee and Hsueh, 2000). In mammals, the original pool of primordial follicles is the only source of all developing follicles for the entire reproductive life. The prerequisite for successful fertilization is the activation of primordial follicles. AMPK protein is reported to be expressed in mammalian ovaries, including oocytes, granulosa cells, theca cells, and corpus luteum (Tosca et al., 2005, 2007a; Lu et al., 2017). A large number of researches show that AMPK is involved in the regulation of primordial follicle activation (Lu et al., 2017). *In situ* ovarian intramural administration of AMPK inhibitor CC in mice, primordial follicle activation and stimulation of follicle growth were observed, and thus more healthy pups were delivered (Lu et al., 2017). AMPK inhibition by CC promotes the growth of cultured ovary *in vitro* by activating mTOR and increasing the expression of *Ctgf* in the YAP signaling pathway (Lu et al., 2017). Importantly, conditional knockout of LKB1 or mTOR inhibitor gene TSC1 in mouse oocytes caused severe subfertility and the pool of primordial follicles exhausted due to early excessive activation (Jiang et al., 2016). In comparison, specific deletion of TSC1 in mouse granulosa cells does not compromise mouse fertility, and the conditional knockout mice can produce more ovulating oocytes (Huang et al., 2013). This phenotype could be explained by increased mTORC1 activity, while rapamycin as a specific mTORC1 inhibitor effectively reversed the condition (Huang et al., 2013; Cheng et al., 2015; Jiang et al., 2016). In addition to using the knockout mouse models *in vivo*, researchers utilized mammalian oocytes by *in vitro* maturation (IVM) technique to investigate the effect of AMPK activation on follicular development. AMPK activation using AICAR or other activators *in vitro* can delay oocyte maturation in swine cumulus-oocyte complexes (COCs), but only in the presence of cumulus cells (Santiquet et al., 2014). Cumulus cells are necessary for promoting the inhibitory effect of AMPK activation on the recovery of swine and bovine oocyte meiosis (Santiquet et al., 2014). During IVM, the level of AMPK phosphorylation gradually decreased in immature bovine oocytes and cumulus cells. The AMPK activator metformin blocks most oocytes in the GV stage by inhibiting the activation of key factors involved in protein synthesis, while CC reduced AMPK phosphorylation in COCs and accelerated the resumption of oocyte meiosis (Tosca et al., 2007b). However, metformin did not inhibit the resumption of denuded oocytes meiosis, suggesting that the inhibition of oocyte maturation by metformin requires the presence of cumulus cells (Tosca et al., 2007b). Communication between the oocyte and cumulus cells is supported by gap junctions, which are important for controlling oocyte maturation. In bovine COCs, AMPK also exists in the gap junctions (Bilodeau-Goeseels et al., 2007). Therefore, the occurrence of oocyte maturation is highly likely to need signals induced by AMPK inhibition from cumulus cells that transmit through gap junctions. Intriguingly, phospho-AMPK provides a meiotic maturation signal and induces oocyte maturation in mice (Downs et al., 2002; Chen et al., 2006). The activation of AMPK not only promotes GVBD in mouse oocytes but also participates in the regulation of the progression of meiosis to MII (Downs et al., 2010; Table 1). More studies will be needed to determine the role of AMPK in follicular development as it appears there are significant differences between species.

**TABLE 1:** Effects of the use of AMPK activator or inhibitor in follicular development.

Compound	Species	Tissue/cell type	*In vivo / In vitro*	Effects	References
Compound C	Mice	Ovary	*In vivo*	Stimulation of follicular development	Lu et al., 2017
AICAR	Swine	COCs	*In vitro*	Inhibition of both oocyte maturation and cumulus cell expansion	Santiquet et al., 2014
Metformin	Bovine	COCs/DOs	*In vitro*	Inhibition of cumulus cell expansion and oocyte arrest at the GV stage/No inhibition of meiotic progression observed	Tosca et al., 2007b
AICAR, Rosiglitazone	Mice	COCs, DOs	*In vitro*	Oocyte maturation	Downs et al., 2002; Chen et al., 2006
AICAR	Mice	CEO, DOs	*In vitro*	Accelerated polar body formation	Downs et al., 2010

*COCs, cumulus-oocyte complexes; DOs, denuded oocytes; CEO, cumulus cell-enclosed oocyte.*

### AMPK in Granulosa Cell Secretion and Proliferation

It is well known that FSH and IGF-I regulate the growth and differentiation of granular cells in mice (Baker et al., 1996; Zhou et al., 1997; Abel et al., 2000). Mice lacking IGF-I had stunted follicular development and were unable to produce Graafian follicles successfully (Baker et al., 1996; Zhou et al., 1997). Adashi et al. (1986) demonstrated that FSH and IGF-I could synergistically act on the MAPK ERK1/2 pathway in granular cells to increase progesterone secretion. In rat and bovine, when primary granulosa cells treated with AICAR or metformin, the activation of AMPK inhibited phosphorylation of MAPK ERK1/2 and affected the expression of 3β-hydroxysteroid dehydrogenase (3β-HSD) and the steroidogenic acute regulatory (StAR) protein, resulting in decreased progesterone secretion (Tosca et al., 2005, 2007a). Besides, metformin can inhibit the proliferation and protein synthesis of bovine granulosa cells through an AMPK-dependent mechanism (Tosca et al., 2006). Similarly, metformin treatment could block the expansion of bovine cumulus cells *in vitro* (Tosca et al., 2007b).

### AMPK in Mammalian Pregnancy Regulation

**FIGURE 3:**
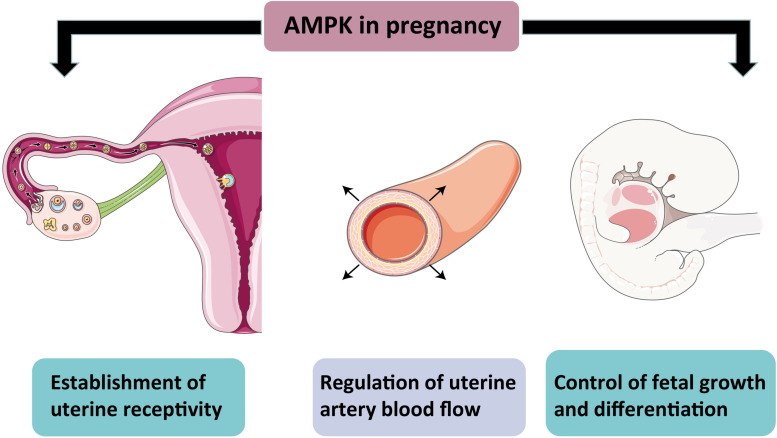
Schematic representation of AMPK action in pregnancy. The diagram shows the importance of AMPK for a normal pregnancy. AMPK activity helps establish uterine receptivity and regulates arterial blood flow and controls normal fetal growth and development.

A healthy fetus delivery demands uterine receptivity increase and normal placenta in the maternal uterus to allow embryo implantation. AMPK is commonly expressed in the all-female reproductive system (McCallum et al., 2018), suggesting that AMPK is essential for normal pregnancy in females. AMPK activation has been shown to reduce fibrosis or scar mature failure after injuring the liver, heart, kidney, intestine, and peritoneum (Noppe et al., 2014; Chen et al., 2017; Cieslik et al., 2017; Shin et al., 2017). Similarly, the conditional deletion of AMPKα in mouse uteri failed scar-free regeneration of the endometrium after delivery and severe endometrial fibrosis, thereby causing embryo implantation failure and lifetime fecundity reduction (McCallum et al., 2018). Another latest report supports the role of AMPK in establishing uterine receptivity and demonstrates that AMPK activity plays a prominent part in epithelial cell proliferation and decidualization (Griffiths et al., 2020). In addition, pharmacological AMPK activation can promote uterine artery vasodilation, which indicates that AMPK activation may help to maintain uteroplacental blood flow, thereby ensuring a normal pregnancy process (Skeffington et al., 2016). AMPK is also required for the growth and differentiation of mammalian embryos. Double knockout of PRKAA1 and PRKAA2 in mice resulted in embryonic lethality around 10.5 days of gestation (Viollet et al., 2009). However, AMPK activation beyond the normal physiological range is detrimental to optimal preimplantation embryo development. Applying AICAR to mouse 2-cell embryos decreases blastocyst formation and inhibits trophectoderm differentiation. AICAR treated embryos displayed altered blastocyst formation gene expression and increased tight junction permeability, which is irreversible. This effect is confirmed with other AMPK activators, metformin and phenformin (Calder et al., 2017). The result suggested that AMPK activity must be tightly controlled to facilitate normal preimplantation development and blastocyst formation. Given that there is no evidence to ensure the harmlessness of metformin for fetal development, the use of metformin during pregnancy should be carefully considered. A study reported that the inhibition of AMPK activity in trophoblast stem cells blocked their normal differentiation under cellular stress (Zhong et al., 2010). After knocking down AMPKα1 and α2 in the mouse trophoblast progenitor cell line SM10, the researchers observed an impaired proliferation and differentiation of progenitor cells (Carey et al., 2014). Glucose is a primary energy source for the growing baby, but after knockdown AMPKα, the glucose transportability in progenitor and differentiated labyrinthine cells decreased. This finding is consistent with a study showing that AMPK regulates Glut3 transporters’ movement toward the plasma membrane and thus increases glucose transport in neurons (Weisova et al., 2009). In summary, AMPK impacts various aspects of normal pregnancy in females, not only as an energy sensor but also participates in the precise regulation of tissue growth and differentiation (Figure 3).

## AMPK in Genital Diseases

### Genital Cancers

The American Cancer Society reported that there were 317, 260 genital cancers cases diagnosed with a mortality rate of 21.3% in 2019 in the United States alone, which seriously endangers human reproductive health (Siegel et al., 2020). Currently, there are many therapeutic regimes for genital malignancy, but recurrence always tends to occur, and the overall prognosis for patients remains still poor. Hence, developing novel molecular targets for cancers is imperatively needed. LKB1 is the product of the tumor suppressor gene STK11. Mutation of LKB1/STK11 leads to PJS, and such patients have an increased risk of several cancers (Hearle et al., 2006). As a classical AMPK upstream kinase, blocking LKB1 activation in rat liver cells significantly abolished AMPK activation (Woods et al., 2003). The close correlation between LKB1 and AMPK reminds us that AMPK may regulate the tumor-suppressive effect of LKB1. Clinically, metformin is often used in combination with other anticancer drugs to reduce cancer incidence and achieve better chemotherapy outcomes (Gadducci et al., 2016), suggesting that AMPK may be involved in carcinogenesis. Several studies have reported that AMPK protein functions in genital system cancers. For example, it is reported that 20% of cervical cancer have LKB1 gene mutation (Wingo et al., 2009). Only those cervical cancer cells harboring intact LKB1-AMPK-mTOR signaling are sensitive to the administration of metformin (Xiao et al., 2012). Higher expression of AMPK correlated with a smaller-size cervical tumor (Choi et al., 2015). Therefore, the above studies showed that the activation of AMPK is critical for retarding cervical cancer cell growth. In addition, AMPK activation could induce apoptosis of ovarian cancer cells and delay endometrial cancer progression by inhibiting the mTOR and/or AKT pathways (Erdemoglu et al., 2009; Xie et al., 2011; Yung et al., 2016), which suggesting AMPK appears to serve as a tumor suppressor and exhibits an anti-cancer effect on genital system cancers. Although the principle how of AMPK plays an anti-cancer effect is far from understood, researches on the role of AMPK in cell-cell junction and cell cycle shed light on its other possible mechanisms. AMPK-mediated regulation of apicobasal polarity establishment and tight junction assembly (Forcet et al., 2005; Zhang et al., 2006; Lee et al., 2007) are important to maintain cell normalization and defect in this process increases tumor cell growth (Aznar et al., 2016). It’s now widely accepted that cell cycle arrest is associated with the anti-tumorigenic effect. AMPK can promote a cell cycle arrest at the level of G1 and G2 by directly phosphorylating and thus stabilizing p53 (Imamura et al., 2001; Jones et al., 2005). P53 is a key to understand how AMPK affects proliferation and its regulation by AMPK also reveals the link between metabolism and cell cycle. However, during tumor development processes, cancer cells are often in a state of metabolic stress (hypoxia, hormone deficiency, or radiochemotherapy, etc.), which activates a stress-response molecule AMPK, inhibiting anabolism and promoting catabolism to provide ATP, helping cancer cell escape the crisis (Park et al., 2009). When androgen-dependent prostate cancer cells are subjected to androgen deprivation or hypoxia, AMPK activation induces autophagy, degrading intracellular organelles, and providing sufficient nutrients for the survival of cancer cells (Chhipa et al., 2010). Thus, AMPK activation-induced autophagy is a protective survival mechanism for androgen-dependent prostate cancer cells in a harsh living environment, promoting prostate cancer cells’ transformation into an androgen-independent phenotype (Chhipa et al., 2011). Although AMPK activity has been repeatedly reported to be related to prostate cancer cell growth and poor prognosis (Park et al., 2009), paradoxically, AMPK activation also induces apoptosis of DU-145 prostate cancer cells (Sauer et al., 2012). Based on the current literature reports, AMPK may have a duplex implication in genital system cancer development. AMPK can either counteract growth-stimulating signaling mediated by mTOR activation or act as a metabolic survival factor in cancer cells, depending on different cancer cell types and other compensations within the cell. Further investigation is needed to answer the question and determine whether AMPK can be as a target for drug therapy of reproductive system related diseases.

### PCOS

Polycystic ovary syndrome (PCOS) is a common gynecological endocrine disease that affects about 6–21% of women in reproductive age and is an important cause of female infertility (Joham et al., 2015). Although the exact pathogenesis remains unclear, PCOS patients are often present with ovarian cortical thickening and multiple immature follicles, accompanied by hyperandrogenic and insulin resistance. Metformin is commonly used clinically to treat PCOS patients, which can reduce the serum progesterone and estrogen concentrations, increase patients’ ovulation, fertilization, and pregnancy rates (Vandermolen et al., 2001). Previously, it was widely believed that metformin, as an insulin sensitizer, exerted its effects by improving insulin resistance in PCOS patients (Morley et al., 2017). However, in recent years, studies have found that metformin is likely to rely on AMPK activation mechanism to participate in the treatment of PCOS. AMPK activators resveratrol and metformin have been shown to treat PCOS in rats, reduce the body and ovary weights, testosterone levels were observed, ameliorated the elevated number of secondary and atretic follicles (Furat Rencber et al., 2018). In a study mentioned above, metformin reduces granulosa cells basal and FSH-stimulated steroid hormone secretion by activating AMPK, which improves the formation of excessive follicular cysts in the ovaries of patients with PCOS and promotes the development of dominant follicles (Tosca et al., 2007a). It is important to note that the endometrium of PCOS patients is also in a state of insulin resistance. Compared to healthy fertile women, the expression of AMPKα and insulin-dependent glucose transporter (GLUT4) decrease in the endometrium of PCOS patients, which are associated with adverse reproductive outcomes. The downstream target of AMPK, myocyte enhancer factor 2A (MEF2A), is involved in the regulation of GLUT4 expression. When AMPK activation increases MEF2A expression, MEF2A is transported to the nucleus and binds to the GLUT4 promoter. Therefore, the application of metformin can increase the expression of GLUT4 in the endometrium of PCOS patients, thereby improving endometrial metabolic function and increasing pregnancy rates (Carvajal et al., 2013).

## Transcription Regulation Roles of AMPK in Reproduction

Transcription regulation is a multistep and complicated process that heavily relies on the interaction between TFs and their DNA recognition sites to modulate the activity of genes (Chen and Rajewsky, 2007). In recent years, the emerging role of AMPK signaling in the control of gene expression through TF regulation has been documented. Indeed, when energy shortage triggers AMPK activation due to exercise/contraction in skeletal muscle, peroxisome proliferator-activated receptor-γ co-activator 1α (PGC1α) and transcription factor EB (TFEB)/transcription factor E3 (TFE3) translocate to the nucleus where they activate mitochondrial genes and metabolic genes such as pathways linked to the expression of glucose transporters, glycolytic enzymes (Mansueto et al., 2017; Markby and Sakamoto, 2020). An excellent review published in 2020 has summarized the transcriptional regulation by AMPK signaling (Sukumaran et al., 2020). And more notably, while significant effort has been given to explore the mechanism of how AMPK impacts gene expression, its involvement in the field of reproduction are less appreciated.

**FIGURE 4:**
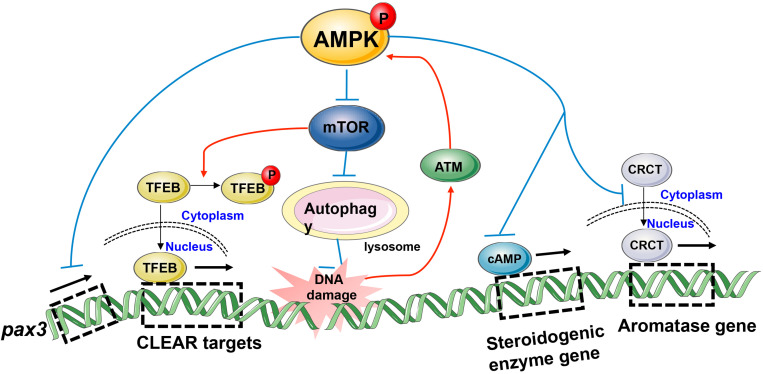
Model depicting transcriptional regulation by phospho-AMPK in the field of reproduction. AMPK activation impacts gene expression involved in biological processes such as embryonic development, spermatocyte DNA damage repair, and gonadal steroid hormones production.

In fact, AMPK can influence reproduction activity not only through acute effects of AMPK activation such as direct phosphorylation of key enzymes but also through controlling slower transcriptional events via modulation of TFs. Both the aforementioned semen cryopreservation and environmental exposure to toxic substances will put spermatozoa under the threat of ROS (Li et al., 2017; Shabani Nashtaei et al., 2017). However, the spermatozoa still retain their capacity of fertilization, indicating spermatozoa themselves have a series of intracellular biochemical reactions to achieve survival in the face of oxidative stress. Increasing evidence has demonstrated that exposure to cadmium (Cd) can result in reproductive toxicity in humans (Pant et al., 2003) and rodents (Li et al., 2016), which is due to Cd-induced ROS accumulation and then DNA damage (Filipic, 2012; Li et al., 2016). Sperm DNA integrity is essential for the accurate transmission of genetic information, and its damage is one of the causes of abnormal sperm and male infertility (Gunes et al., 2015). It has been reported that ATM was activated by Cd-induced DNA damage and then positively regulates autophagy through repression of mTOR signaling via the AMPK pathway in mouse spermatocytes (Li et al., 2017). Subsequently, autophagy provided energy for DNA damage repair via selective degradation of damaged molecules and organelles (Towers and Thorburn, 2016) and thus had a protective role in DNA stability. *In vivo*, AMPK and mTOR always drive autophagy in opposite directions. AMPK facilitates autophagy directly by phosphorylating autophagy-related ULK1 and PIK3C3/VPS34 complexes or regulating the nuclear translocation of autophagy-related transcription factors such as ACSS2 and FOXO3, TFEB, while mTOR1 has an opposite, inhibitory role in these processes (Li and Chen, 2019), thereby maintaining the dynamic balance of autophagy. Actually, autophagy is a catabolic process mediated by lysosomes (Towers and Thorburn, 2016). A paper published by Young et al. (2016) linked AMPK and lysosomal biogenesis to germ cell specification, revealing the possible mechanism by which AMPK determines cell fates. Embryonic stem cells (ESCs) possess the capability to differentiate into multiple cell types (Young et al., 2016). The spatial and temporal integration of numerous signaling cascades govern the induction of lineage-specific transcription factors and determine cell differentiation (Gadue et al., 2006). The transcription factors, TFEB and TFE3, are known as important regulators of lysosomal biogenesis and autophagic processes, whose activation induces Coordinated Lysosomal Expression and Regulation (CLEAR) target genes and up-regulates endolysosomes, thus providing a suitable microenvironment for the canonical Wnt pathway to free β-catenin and controlling the endodermal differentiation program (Young et al., 2016). Mouse ESCs lacking AMPK displayed severe endodermal defects because increased mTOR signaling inhibits TFEB activity, indicating the important role of AMPK in cell fate determination during differentiation (Young et al., 2016). Interestingly, two teams recently reported that AMPK could dephosphorylate TFEB/TFE3, induce their nuclear localization independently of mTOR activity (Collodet et al., 2019; El-Houjeiri et al., 2019), and activate TFEB/TFE3 boost the expression of lysosomal and inflammatory genes in macrophages in response to infection (Hipolito et al., 2018; El-Houjeiri et al., 2019). Additionally, stimulation of AMPK could disturb the expression of specific developmental control genes such as the pax3 gene in the embryo and cause durable changes in phenotype under oxidative stress (Wu et al., 2012). Hence, it is hardly difficult to see that AMPK is involved in a wide variety of biological processes, including the biosynthesis regulation of sex hormones. AMPK activation, whether the use of activators or overexpression of the constitutively active form, inhibits cyclic AMP (cAMP) -mediated steroidogenic enzyme promoter activities and gene expression, ultimately reducing testosterone production in Leydig cells (Ahn et al., 2012). The importance of testosterone in maintaining male fertility is indisputable, while the role of estradiol should not be ignored. The aromatase enzyme in testis irreversibly converts androgens into estrogens, and the infertile aromatase KO mice support the direct actions of estrogen on spermatogenesis (Robertson et al., 2001). A study using human testicular tissues has demonstrated that the expression pattern of pAMPK was inversely associated with aromatase expression and AMPK phosphorylation inhibits the nuclear translocation of the cAMP response element-binding protein- regulated transcription co-activator (CRCT), resulting in failed aromatase expression (Ham et al., 2016), indicating the important role of AMPK in the estrogen-mediated development of germ cells. Taken together, these observations lead to the conclusion that AMPK influences the binding between TFs and their target DNA sequences, thus affecting gene expression in the field of reproduction (Figure 4).

## Conclusion

A large body of research has shown that AMPK is not limited to serve as a major energy sensor to monitor the supply of nutrients, but also involved in the regulation of reproduction through several different strategies. AMPK affects the secretion of GnRH and gonadotropins and integrates energy status with fertility at the level of the hypothalamus and pituitary. Furthermore, AMPK itself is also expressed in the gonad, and is involved in regulating spermatogenesis and follicular development. Of particular concern is the emerging role of AMPK in transcriptional regulation, in which AMPK alters the transcription efficiency of reproduction-related genes by affecting the binding of transcription factors to their specific target sequences. Here, we briefly summarized the effects of AMPK activation on gene expression involving biological processes such as embryonic development, spermatocyte DNA damage repair and gonadal steroid hormone production, hoping to engender interest in the field to fully understand how reproduction and metabolism are tightly connected in the body through transcriptional regulation by AMPK activation.

## Author Contributions

WY and LW drafted the manuscript. FW and SY revised the manuscript. All authors approved the final version of the manuscript.

## Conflict of Interest

The authors declare that the research was conducted in the absence of any commercial or financial relationships that could be construed as a potential conflict of interest.
